# Teleradiology as a relevant indicator of the impact of COVID-19 pandemic management on emergency room activities: a nationwide worrisome survey

**DOI:** 10.1186/s13244-021-00964-0

**Published:** 2021-03-03

**Authors:** Jordan Vatele, Stéphanie Gentile, Vivien Thomson, Bénédicte Devictor, Marine Cloux, Nicolas Girouin, Flavie Bratan, Jean-François Bergerot, Mylène Seux, Nathan Banaste, Karim Tazarourte, Guillaume Gorincour

**Affiliations:** 1 Imadis Teleradiology, Lyon, Bordeaux, Marseille, France; 2 Department of Radiology, Hospices Civils de Lyon, Lyon, France; 3 Faculté de Médecine - Secteur Timone, EA 3279: CEReSS -Centre d’Etude et de recherche sur les Services de Santé et la Qualité de Vie, Aix-Marseille Université, Marseille, France; 4 Ramsay Générale de Santé, Clinique de la Sauvegarde, Lyon, France; 5 DeepLink Medical, Lyon, France; 6 Norimagerie, Caluire et Cuire, France; 7 Department of Diagnostic and Interventional Imaging, Centre Hospitalier Saint-Joseph Saint-Luc, Lyon, France; 8 Ramsay Générale de Santé, Clinique Convert, Bourg-en-Bresse, France; 9 Department of Radiology, Hopital Nord-Ouest, Villefranche-sur-Saône, France; 10 Emergency Department, CHU Edouard Herriot, Hospices Civils de Lyon, Lyon, France; 11 EA 7425 HESPER, Université Lyon 1, Lyon, France; 12 ELSAN, Clinique Bouchard, Marseille, France

**Keywords:** Teleradiology, COVID-19, Emergency service, Hospital, Health services accessibility

## Abstract

**Objectives:**

To evaluate the impact of COVID-19’s lockdown on radiological examinations in emergency services.

**Methods:**

Retrospective, multicentre analysis of radiological examinations requested, via our teleradiology network, from 2017 to 2020 during two timeframes (calendar weeks 5–8 and then 12–15). We included CT scans or MRIs performed for strokes, multiple traumas (Body-CT), cranial traumas (CTr) and acute non-traumatic abdominal pain (ANTAP). We evaluated the number and percentages of examinations performed, of those with a pathological conclusion, and of examinations involving the chest.

**Results:**

Our study included 25 centres in 2017, 29 in 2018, 43 in 2019 and 59 in 2020. From 2017 to 2019, the percentages of examinations were constant, which was also true for chest CTs. Each centre’s number of examinations, gender distribution and patient ages were unchanged. In 2020, examinations significantly decreased: suspected strokes decreased by 36% (1052 vs 675, *p* < 0.001), Body-CT by 62% (349 vs 134, *p* < 0.001), CTr by 52% (1853 vs 895, *p* < 0.001) and for ANTAP, appendicitis decreased by 38% (45 vs 90, not statistically significant (NS)) sigmoiditis by 44% (98 vs 55, NS), and renal colic by 23% (376 vs 288, NS). The number of examinations per centre decreased by 13% (185.5 vs 162.5, *p* < 0.001), whereas the number of examinations of the "chest" region increased by 170% (1205 vs 3766, *p* < 0.001).

**Conclusion:**

Teleradiology enabled us to monitor the impact of the COVID-19 pandemic management on emergency activities, showing a global decrease in the population's use of care.

## Key points

- During the first weeks of lockdown, a decreased flow of trauma management was observed (and expected) for Body-CT and cranial trauma, due to travel restrictions.
- However, a significant decrease in non-traumatic consultations was also noted (stroke, ANTAP). Many sick patients (stroke, appendicitis, renal colic, and sigmoiditis) did not consult the emergency department services, which suggests a decrease in the use of care by the population.
- Teleradiology, because of its capacity to manage numerous ER spread over the territory, is a reliable and early tool for analysing variations in the practice of ERs. Measures to inform the general public must be taken to avoid repetition of this situation, as the second COVID-19 wave is happening.

## Introduction

The coronavirus epidemic appeared on November 17, 2019 in the city of Wuhan (Hubei province) in China. The virus quickly spread around the world, with Europe being particularly impacted at the outset of the pandemic. The first cases were recorded in France as of January 24, 2020.

Barrier measures were settled to prevent exponential spread. The French population’s lockdown was decreed on March 17, 2020, and continued until May 11, 2020.

Contained was meant, in part, to avoid overloading the health care system, especially in intensive care. The dramatic decrease in travel and other activities resulted in significant life style changes in the French population.

The fear of contracting the virus, the reduction in public transportation, and the overloaded health facilities were elements which impacted access to the health care system, and in particular the use of the emergency room (ER) [1].

Several studies showed that, during this period, the use of health care systems was reduced [2] due to a phenomenon of postponing care [3] and the lessened accessibility and availability of care.

Intuitively, a decline was anticipated in the number of trauma patients seen as a result of the restricted movements during lockdown. Some recent studies, however, have shown a decrease in activity and/or delays in treatment for the management of cardio-vascular [4], neuro-vascular [5] and psychiatric disorders [6].

Besides, from a radiological standpoint, as evoked by Boeken et al. in a short report, there was sharp drop in non-COVID-19-related CTs during the current health crisis compared with a similar period in 2019 [7].

Thus, assessing the impact of lockdown on a certain number of emergency sectors through the use of teleradiology appeared meaningful.

Although indirect, the use of teleradiology data is reliable because it reflects the activities of emergency room services [8, 9]. In addition, the teleradiological organization makes it possible to cover a large territory and therefore obtain a global vision of the emergency care activity in a wide area. Emergency teleradiology companies in France, whether private or public, remotely interpret all CT and MR requested by ER departments of partner hospitals during night-shift hours (usually from 6 pm to 8 am), in partial or complete collaboration with hospitals' own radiologists. In our organization, every single request is medically validated by one of the on-call teleradiologist (TR) called the regulator, and all TRs work in dedicated centres with specific high-standard ergonomic conditions. It is noteworthy that in France, radiologists cannot be exclusively TRs and are obliged to maintain a predominant clinical onsite workload.

Our study’s aim was to evaluate, in a retrospective and multicentre study, the qualitative and quantitative impact of lockdown following the COVID-19 pandemic on the use of emergency services for cerebrovascular accidents (CVA), polytrauma and head trauma, as well as acute non-traumatic abdominal pain. To do this, we compared the teleradiological activity before and during the confinement period, over the last 4 years.

## Material and methods

### Selection of periods studied

Our analysis focused on two periods:

Period 1 focused on calendar weeks 5–8 of the years 2017, 2018, 2019, 2020.

Period 1 included four classic winter weeks in France and excluded school holidays and was considered our reference period.

Period 2, centred on calendar weeks 12–15 of the years 2017, 2018, 2019, 2020 which included the first 4 weeks of lockdown decided by the French government in 2020.

### Selection of the centres studied

Our 59 French hospital teleradiology partners were included for all the study periods.

In order to remain within each centre’s scope of services, only the centres who were partners since week 5 of each year were taken into account. The number of centres for each period was collected.

For each centre, we identified its geographic area and classified the regions according to Public Health France map reflecting mortality during the pandemic.

### Selection of sectors studied

Our teleradiology structure has an exclusive emergency imaging activity. The ITIS teleradiology tool (Deeplink medical, Lyon, France) classifies parts of our activities into clinical sectors. Since our activity began, some of these sectors have been documented and monitored, as they serve as indicators for the support team and partner centres [10]. These included cerebrovascular accidents (CVA), cranial trauma (CTr), and Body-CTs for multiple trauma and acute non-traumatic abdominal pain (ANTAP) courses.

Except for those with traumatic abdominal pain, all the patients referred in these four sectors over the selected periods were included.

Examinations classified as being pathological were based on the following criteria:

- *CVAs* recent ischemic or hemorrhagic brain injury, significant supra-aortic arterial lesion (stenosis > 50%, dissection, occlusion) or intracranial vascular abnormality (stenosis, occlusion).
- *Body-CT* one or more post-traumatic lesions.
- *CTr* intracranial bleeding or craniofacial fracture.
- *Acute non-traumatic abdominal pain* diagnosis of acute appendicitis, sigmoiditis or renal colic.

The anatomical regions of all performed examinations can also be analyzed (skull, thorax, abdomen, and pelvis): particular attention was paid to examinations of the "chest” region.

### Variables studied

All the data used in this study came from the teleradiological database, and all patient anonymity was guaranteed.

The total number of examinations per period was collected (as well as the average number of examinations per centre) overall and for each sector as well as for the “chest" anatomical region. The percentage of examinations in each sector was calculated, as well as the percentage of positivity (pathological results for each sector).

The age, sex, patient’s region of origin, were collected globally and in the four sectors (except for the anatomical region “chest” where this data collection was global).

The evolution rates in examination requests between time periods were compared between different subgroups by region.

**Fig. 1:**
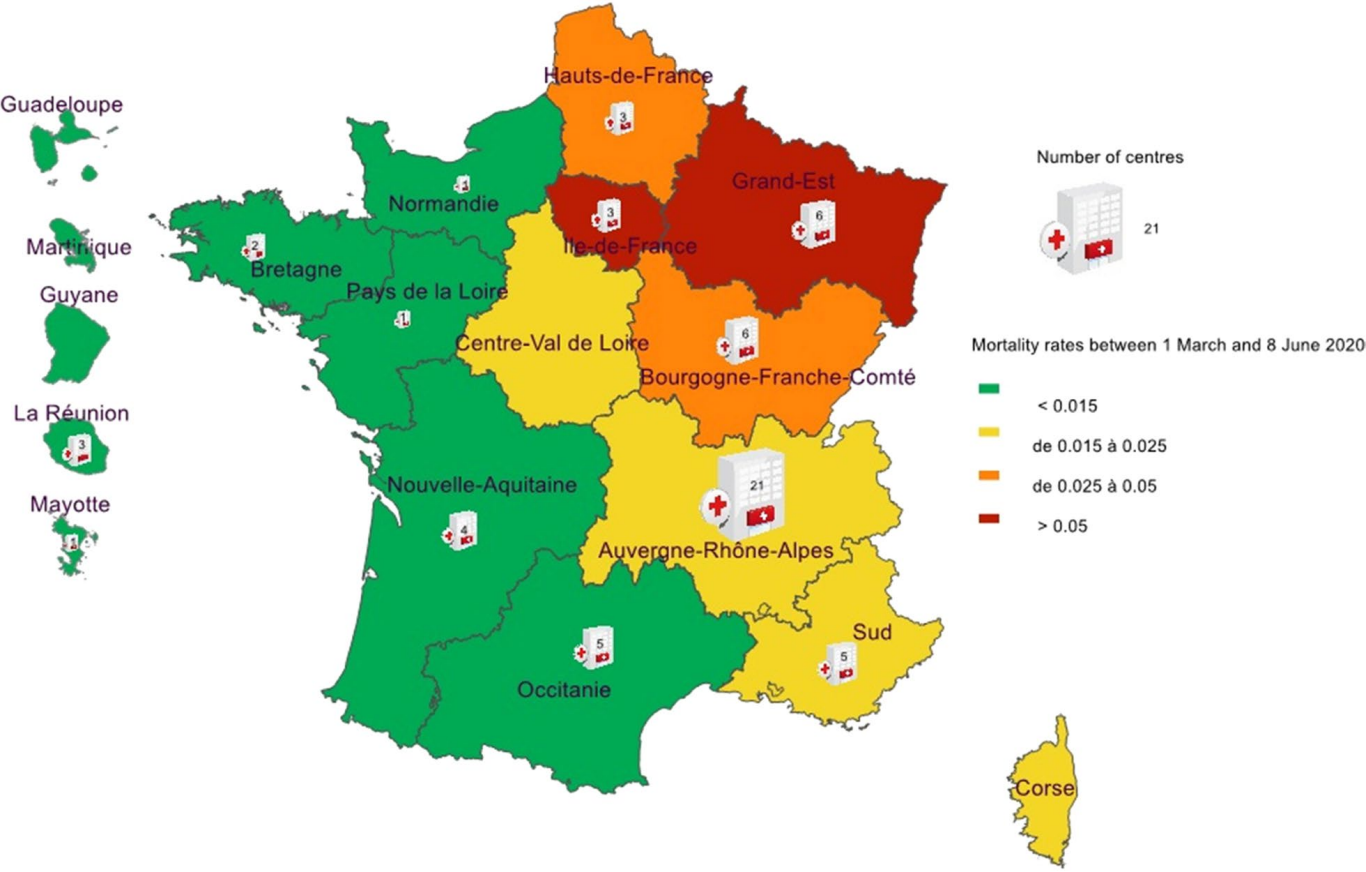
Number of centres and distribution of mortality by each French region

In Fig. 1, regions have been represented according to mortality due to COVID-19 between March 1 and June 8, 2020, and the discretization for the color classes of the regions was done according to the Jenks Index.

The Jenks Index synthesizes the homogeneity of values within each class, and the heterogeneity of classes between them. Partner centres are also shown.

### Statistical analysis

Statistical analyses were carried out using SPSS version 20 software. The dichotomous variables were described as whole integers and percentages, and the continuous variables as mean and standard deviation. The distribution of all variables was analyzed with the Kolmorogov–Smirnov test. The data were compared between years, and according to the pre- and per-containment periods. By the Chi 2 test. A Student Test or Analysis of Variance (ANOVA) was performed for the quantitative variables. The significance level adopted was 5%.

Our study was approved by the Ethics Committee of the College of French Radiology Academics (IRB CRM-2007-108).

## Results

There were 25 centres included in 2017, 29 centres in 2018, 43 in 2019 and 59 in 2020.

The distribution of centres is shown in Fig. 1 and superimposed on the mortality during the pandemic.

In 2017, 2018 and 2019, between periods 1 and 2, the percentage of examinations per sector was constant. The same was true for the percentage of examinations of the chest region. The number of examinations per centre was the same in all periods for the years 2017 to 2019, as was the proportion of males, females and of the mean age (Table 1).

**Table 1:** Characteristics of the study population

	2017-Period 1	2017-Period 2	2018-Period 1	2018-Period 2	2019-Period 1	2019-Period 2	2020-Period 1	2020-Period 2	*p* value
Centres (*n*)	25	25	29	29	43	43	59	59	
**Total examinations (n)**	3291	3321	4242	4491	7265	7383	10,944	9576	
Examinations per centres (*n*)	131.64	132.84	146.27	154.86	168.95	171.69	185.49	162.30	
**CVA sector (n)**	449	456	544	601	921	909	1052	675	
Age—mean	67 ± 17.9	65.7 ± 18.3	67.6 ± 18.3	69.8 ± 18.9	67.6 ± 18.7	66.6 ± 18.6	66.9 ± 18.5	68.5 ± 17.7	
Gender—Male (*n* (%))	211 (47%)	224 (49%)	251 (46%)	277 (46%)	431 (47%)	432 (47%)	498 (48%)	345 (52%)	
Pathological CVA (*n* (%))	97 (22%)	93 (20%)	126 (23%)	131 (22%)	177 (19%)	203 (22%)	275 (25%)	190 (27%)	
**CTr Sector (n)**	593	565	771	817	1310	1330	1853	895	
Age—Mean	59 ± 27.4	55.1 ± 29.2	59.6 ± 21.2	60.15 ± 27.7	62.2 ± 27.2	61.4 ± 27.7	63.41 ± 27.6	66.3 ± 25.5	
Gender—Male (*n* (%))	335 (56%)	314 (55%)	427 (55%)	458 (56%)	714 (54%)	754 (56%)	1001 (55%)	514 (57%)	
Pathological CTr (*n* (%))	99 (17%)	105 (19%)	125 (16%)	116 (14%)	201 (15%)	230 (1%)	305 (17%)	143 (15%)	
**Body-CT Sector (n)**	98	121	97	149	208	220	349	134	
Age—Mean	35.7 ± 18.4	35.8 ± 18.5	40 ± 18.7	37.4 ± 20.2	37.9 ± 20.2	36.9 ± 19.4	37.6 ± 21.5	41.1 ± 19.6	
Gender—Male (*n* (%))	72 (73%)	93 (76%)	70 (72%)	105 (70%)	152 (73%)	150 (68%)	235 (66%)	102 (80%)	
Pathological BC (*n* (%))	59 (60%)	71 (59%)	51 (53%)	79 (53%)	97 (47%)	103 (47%)	171 (48%)	80 (60%)	
**ANTAP Sector (n)**	998	1036	1277	1416	2251	2239	3523	2507	
Age—Mean	56.15 ± 21.45	54.5 ± 21.2	57.8 ± 21.1	56.5 ± 21.5	54.6 ± 21.86	58.7 ± 21.9	**57.7 ± 22.7**	**59.61 ± 21**	**0.001**
Gender—Male (*n* (%))	519 (52%)	523 (51%)	644 (50%)	695 (49%)	1116 (50%)	1119 (48%)	1782 (51%)	1324 (53%)	NS
Acute appendicitis (*n*)	45	54	49	56	73	86	145	90	
Sigmoiditis (*n*)	34	31	41	38	71	86	98	55	
Renal colic CCN (*n*)	119	130	153	169	246	232	376	288	
Pathological ANTAP* (%)	20%	21%	19%	21%	17%	18%	17	18%	
**Chest region (n)**	306	306	490	463	816	788	1205	3766	

NB: A summary of the most relevant comparisons will appear in Table 2

*CVA:* cerebrovascular accidents, *CTr:* cranial trauma, *Body-CT:* Body-CTs for multiple trauma, *ANTAP:* acute non-traumatic abdominal pain, *NS:* not statistically significant

*Pathological ANTAP = 3 cumulative diseases (Acute appendicitis + Sigmoiditis + Renal colic)

In 2020, except for the age of the patients, the patients’ sociodemographic characteristics were not statistically different between Periods 1 and 2 for all the sectors. It was noted that the patients’ ages were higher in the ANTAP sector during the period 2 (57.7 years ± 22.7 in period 1 vs 59.6 years ± 21 in period 2, *p* < 0.001).

**Fig. 2:**
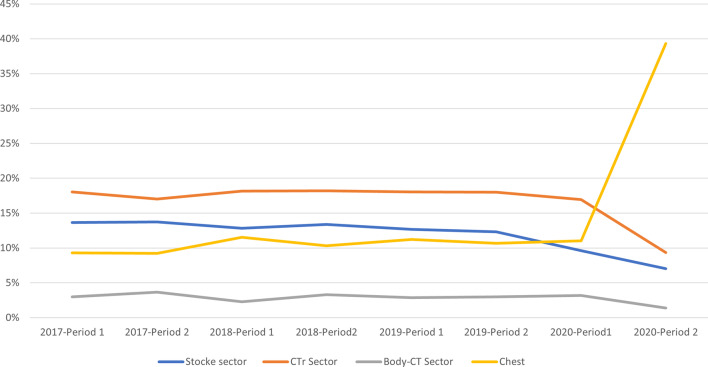
Evolution of the studied sectors and examinations of the chest region over each period. *Stroke sector = CVA sector:* cerebrovascular accidents, *CTr:* cranial trauma, *Body-CT:* Body-CTs for multiple trauma, *ANTAP:* acute non-traumatic abdominal pain

By contrast, in 2020, the numbers and percentage of examinations were different between the two periods, with a decrease for all the sectors. However, examinations of the anatomical “chest” region increased significantly (Fig. 2).

In 2020, 10,944 examinations (i.e. 185.5 examinations per centre) were interpreted during Period 1 as compared to 9576 examinations (i.e. 162.3 examinations per centre) during Period 2. Data analysis according to the region of origin of the partner centres, and in particular those regions most affected by COVID, did not show significant differences in the number, percentage and type of examinations performed.

Regardless of the year, period and sector, the proportion of so-called pathological examinations was the same.

**Fig. 3:**
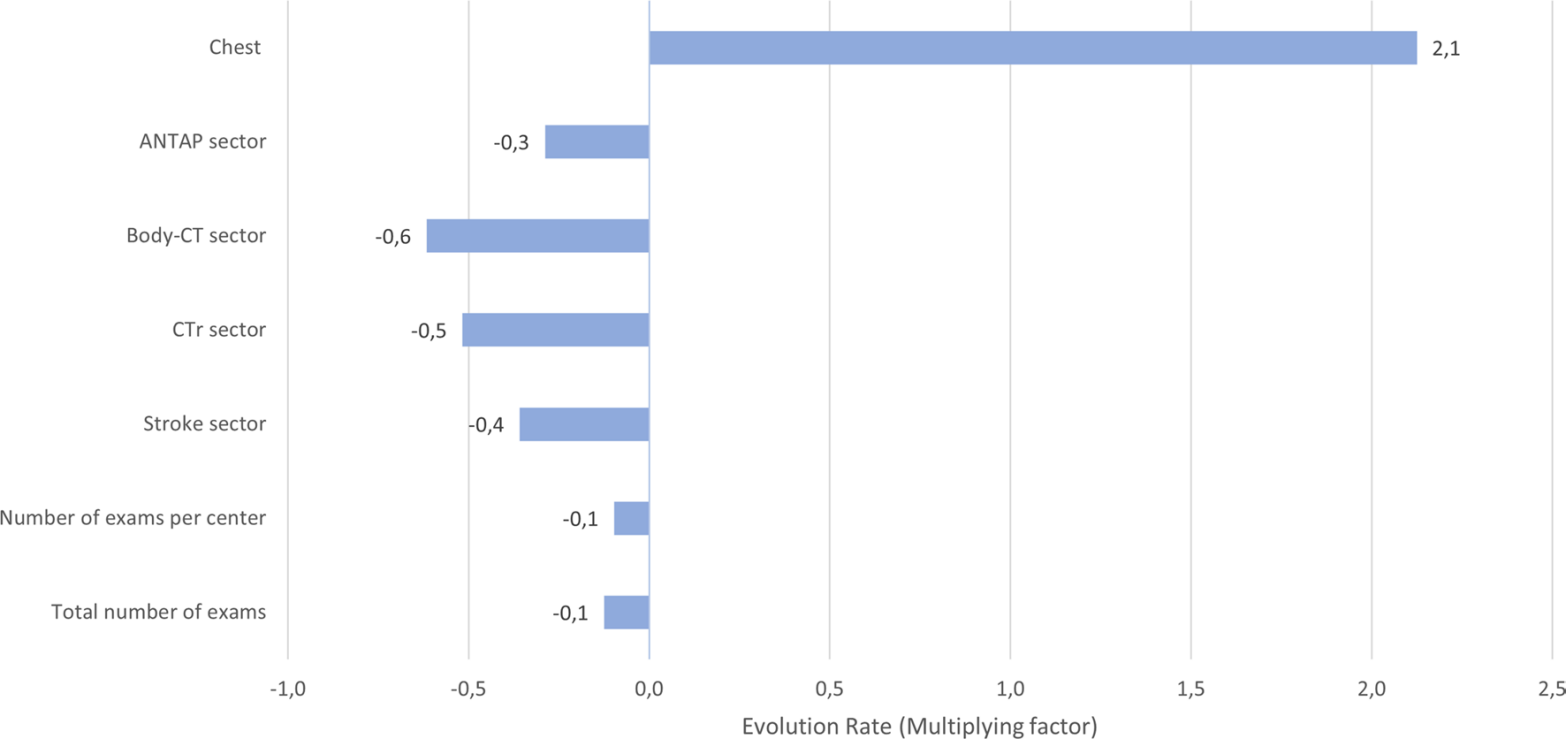
Periods 1 and 2 rate of change (on the *x*-axis multiplying factor) in 2020 in the number of examinations per sector, for the thoracic region and per centre. *Stroke Sector = CVA Sector:* cerebrovascular accidents, *CTr:* cranial trauma, *Body-CT:* body-CTs for multiple trauma, *ANTAP:* acute non-traumatic abdominal pain

The rate of change in the number of examinations in each sector is shown in Table 2 and Fig. 3.

**Table 2:** Number of overall examinations and by sector over all the periods studied

	2017-Period 1	2017-Period 2	2018-Period 1	2018-Period 2	2019-Period 1	2019-Period 2	2020-Period 1	2020-Period 2
Total examinations (n)	3291	3321	4242	4491	7265	7383	10,944	9576
CVA Sector	449 (14%)	456 (14%)	544 (13%)	601 (13%)	921 (13%)	909 (12%)	1052 (10%)	675 (7%)*
CTr Sector	593 (18%)	565 (17%)	771 (18%)	817 (18%)	1310 (18%)	1330 (18%)	1853 (17%)	895 (9%)*
Body-CT Sector	98 (3%)	121 (4%)	97 (2%)	149 (3%)	208 (3%)	220 (3%)	349 (3%)	134 (1%)*
Chest	306 (9%)	306 (9%)	490 (12%)	463 (10%)	816 (11%)	788 (11%)	1205 (11%)	3766 (39%)*
ANTAP Sector	998 (30%)	1036 (31%)	1277 (30%)	1416 (32%)	2251 (31%)	2239 (30%)	3523 (32%)	2507 (26%)*

*CVA: Cerebrovascular accidents; CTr: Cranial trauma; Body-CT: Body-CTs for multiple trauma; ANTAP: Acute non-traumatic abdominal pain*

(*) Significant differences between periods 1 and 2 of 2020, p < 0.001

Thus,

- the stroke sector decreased by 36% (1052 vs 675, *p* < 0.001)
- the body-CT sector decreased by 62% (349 vs 134, *p* < 0.001)
- the CTr sector decreased 52% (1853 vs 895, *p* < 0.001)
- the number of examinations per centre decreased by almost 13% (185.5 vs 162.5, *p* < 0.001)
- the number of examinations of the "chest" region increased by nearly 170% (1205 vs 3766, *p* < 0.001)

The rate of change in the main diagnoses of the ANTAP sector showed a trend towards a decrease in all three targeted pathologies.

- Appendicitis decreased by 38% (145 vs 90, NS),
- Sigmoiditis decreased by 44% (98 vs 55, NS),
- Renal colic decreased by 23% (376 vs 288, NS).

## Discussion

Due to COVID-19 outbreak, The French president eventually decreed a national lockdown on March 16, 2020, i.e. at the beginning of week 12.

The primary interest of our study is to confirm, through a cohort of more than 50 centres, the significant decrease of radiological procedures in ER [7], during the COVID lockdown, especially for trauma, suspicion of stroke, appendicitis, renal colic and sigmoiditis.

In our series, all the examinations (except those concerning the "chest" region) showed a significant decrease during lockdown.

In particular, the number of stroke examinations decreased by 36%, which is similar to the data from the study by Kansagra et al. (39%) [11].

By contrast, the decrease in our study was greater than that found in Kerleroux et al. study [5] which showed a 21% decrease in thrombectomies. This may suggest that patients with minor or regressive symptoms had less options to access emergency services.

According to the Santé Publique France report of 2020 calendar week 16, the number of emergency department visits and hospitalizations for stroke decreased by approximately 27% as compared to the same period in 2019 [2].

Concerning ANTAPs, the number of examinations decreased by 29% for no apparent reason, and for renal colic, the decrease in the number of examinations could be related to codified and well-known medical treatments which can be more easily managed on an outpatient basis. Additionally, its recurrence is frequent and patients recognize the symptoms without having to go to an emergency department. For appendicitis and sigmoiditis, a clinical presentation without signs of severity may have gone unnoticed or been medically treated by their general practitioner.

Indeed, several articles show that non-surgical treatment of acute appendicitis is an effective and reasonable approach for certain groups of patients and has no resultant increase in the number of complications [12–14].

For the CTr and Body-CT sectors, the number of examinations decreased by 52% and 62%, respectively. This decrease was predictable due to an almost complete cessation of outdoor activities, travel and associated car accidents. These figures corroborated with the road safety indicators of March 2020 which announced 43.2% fewer injury accidents as compared to March 2019.

Our results showed a worrisome overall drop in the number of examinations in the stroke and ANTAP sectors over a large part of France during the first 4 weeks of lockdown. Since we experienced similar decreases in normal and pathological examinations (stable percentage of positivity), the relevance of examinations was not higher. This does not prejudge the severity of patients with a pathological examination, and we were not able to specifically study this severity patterns.

Our study shows that patients did not access emergency room services, either renouncing or having no access to care (unavailability of medical referents and/or misdirection of the emergency call centres by misunderstanding the patients’ complaints). This situation can have dramatic effects because the access to emergency department services is necessary for these patients.

This phenomenon can be explained by several factors such as the patient’s overriding fear of exposure to COVID-19, despite their medical symptoms. This phenomenon also occurred during the 2003 SARS epidemic in Asia when there was a recorded average reduction of 30–50% in all non-critical emergency department visits [15–17].

Additionally, the pandemic’s resultant strain on emergency department facilities and/or closure of many doctors’ office, along with reduced public transportation added to the difficulty of accessing emergency department services [18]. This phenomenon no doubt was exacerbated by policy recommendations to avoid disturbing the medical staff who were already overburdened by the pandemic.

In the weeks following the end of lockdown, we will need to document the return to using emergency department services, the resultant delay in treatments, increased sequelae and increased cardiovascular mortality.

In order to anticipate and document this phenomenon during any future lockdown, it will be important to establish and follow indicators so as to reinforce the public health messages for other medical problems (stroke in particular) concomitantly with those related to the epidemic. Additionally, the health care system will need to adapt and the private and public sectors will have to strengthen their collaboration. For example, separating “pandemic” hospitals/clinics from clean hospitals/clinics could be an appropriate solution, to be dealt with in a territorial perspective. Besides, the development of telemedicine/teleconsultations solutions will help maintain access to care for appropriate medical conditions.

The significant increase in the tests involving the "chest" region directly reflects the peak of the outbreak and the importance of diagnostic CT scans [19]. This requires a major reorganization of radiological structures in general and teleradiological structures in particular.

Expertise in chest imaging varies and, in order to maintain, or even improve diagnostic performances, specific training actions must also be anticipated.

Our study contains the biases of a retrospective multi-centre data collection. However, the extent of recruitment on French territory and the homogeneity of the data collected through our specific and structured sectors reinforces the accuracy of our results.

Teleradiology appears to be a relevant indirect indicator of the impact of the COVID-19 pandemic management on the activities of emergency department services.

## Conclusion

Thanks to teleradiology, we have been able to confirm initial brief report by Boeken [7] in a wider and more structured approach, and to follow, in an indirect but structured manner, the impact of the COVID-19 pandemic management on the activities of a large cohort of emergency room services.

During the first weeks of lockdown, a decreased flow of trauma management was observed (and expected) for Body-CT and cranial trauma, due to travel restrictions.

However, a significant decrease in non-traumatic consultations was also noted (stroke, ANTAP). Our study shows that many sick patients (stroke, appendicitis, renal colic, and sigmoiditis) did not consult the emergency department services, which suggests a decrease in the use of care by the population.

Several explanations for this phenomenon can be put forth, such as fear of contracting the virus, restricted freedom of movement, difficulties in accessing the health system and the desire to avoid disturbing an already overloaded medical staff.

The reduction in the use of patient care, reflected in the dramatic drop in radiological procedures, will probably lead to a risk of loss of opportunity and a potential negative prognostic impact.

In addition, teleradiology, because of its capacity to manage numerous ER spread over the territory, is a reliable and early tool for analysing variations in the practice of ERs.

Measures to inform the general public must be taken and everything possible must be done to avoid a repetition of such a situation, as the second COVID-19 wave is now happening.

## Abbreviations

ANTAP: Acute non-traumatic abdominal pain
Body-CT: Whole-body computed tomography
COVID-19: Coronavirus disease 2019
CT: Computed tomography
CTr: Cranial traumas
CVA: Cerebrovascular accidents
ER: Emergency room
MRI: Magnetic resonance imaging
NS: Not statistically significant
SARS: Severe acute respiratory syndrome
TR: Teleradiologist

## Availability of data and material

The datasets used and/or analyzed during the current study are available from the corresponding author on reasonable request.
